# Monitoring retinal changes with optical coherence tomography predicts neuronal loss in experimental autoimmune encephalomyelitis

**DOI:** 10.1186/s12974-019-1583-4

**Published:** 2019-11-04

**Authors:** Andrés Cruz-Herranz, Michael Dietrich, Alexander M. Hilla, Hao H. Yiu, Marc H. Levin, Christina Hecker, Andrea Issberner, Angelika Hallenberger, Christian Cordano, Klaus Lehmann-Horn, Lisanne J. Balk, Orhan Aktas, Jens Ingwersen, Charlotte von Gall, Hans-Peter Hartung, Scott S. Zamvil, Dietmar Fischer, Philipp Albrecht, Ari J. Green

**Affiliations:** 1 Division of Neuroimmunology and Glial Biology, Department of Neurology, University of California, San Francisco, San Francisco, USA; 2 Department of Neurology, Medical Faculty, Heinrich-Heine University, Düsseldorf, Germany; 3 Department of Cell Physiology, Faculty of Biology and Biotechnology, Ruhr-University Bochum, Bochum, Germany; 4 Department of Ophthalmology, University of California, San Francisco, San Francisco, USA; 5 Department of Ophthalmology, Palo Alto Medical Foundation, Palo Alto, CA USA; 6 Institute of Anatomy II, Medical Faculty, Heinrich-Heine University, Düsseldorf, Germany; 7 Department of Neurology, Klinikum rechts der Isar, Technical University of Munich, Munich, Germany; 8 Department of Neurology, Amsterdam UMC, Vrije Universiteit Amsterdam, Amsterdam, The Netherlands; 9 Program in Immunology, University of California, San Francisco, San Francisco, USA

**Keywords:** Experimental autoimmune encephalomyelitis, Experimental optic neuritis, Optical coherence tomography, Optokinetic response, Multiple sclerosis, Neurodegeneration

## Abstract

**Background:**

Retinal optical coherence tomography (OCT) is a clinical and research tool in multiple sclerosis, where it has shown significant retinal nerve fiber (RNFL) and ganglion cell (RGC) layer thinning, while postmortem studies have reported RGC loss. Although retinal pathology in experimental autoimmune encephalomyelitis (EAE) has been described, comparative OCT studies among EAE models are scarce. Furthermore, the best practices for the implementation of OCT in the EAE lab, especially with afoveate animals like rodents, remain undefined. We aimed to describe the dynamics of retinal injury in different mouse EAE models and outline the optimal experimental conditions, scan protocols, and analysis methods, comparing these to histology to confirm the pathological underpinnings.

**Methods:**

Using spectral-domain OCT, we analyzed the test-retest and the inter-rater reliability of volume, peripapillary, and combined horizontal and vertical line scans. We then monitored the thickness of the retinal layers in different EAE models: in wild-type (WT) C57Bl/6J mice immunized with myelin oligodendrocyte glycoprotein peptide (MOG_35–55_) or with bovine myelin basic protein (MBP), in TCR^2D2^ mice immunized with MOG_35–55_, and in SJL/J mice immunized with myelin proteolipid lipoprotein (PLP_139–151_). Strain-matched control mice were sham-immunized. RGC density was counted on retinal flatmounts at the end of each experiment.

**Results:**

Volume scans centered on the optic disc showed the best reliability. Retinal changes during EAE were localized in the inner retinal layers (IRLs, the combination of the RNFL and the ganglion cell plus the inner plexiform layers). In WT, MOG_35–55_ EAE, progressive thinning of IRL started rapidly after EAE onset, with 1/3 of total loss occurring during the initial 2 months. IRL thinning was associated with the degree of RGC loss and the severity of EAE. Sham-immunized SJL/J mice showed progressive IRL atrophy, which was accentuated in PLP-immunized mice. MOG_35–55_-immunized TCR^2D2^ mice showed severe EAE and retinal thinning. MBP immunization led to very mild disease without significant retinopathy.

**Conclusions:**

Retinal neuroaxonal damage develops quickly during EAE. Changes in retinal thickness mirror neuronal loss and clinical severity. Monitoring of the IRL thickness after immunization against MOG_35–55_ in C57Bl/6J mice seems the most convenient model to study retinal neurodegeneration in EAE.

## Introduction

Optic neuritis (ON) is an acute, inflammatory demyelinating disease of the optic nerve resulting in impairment of vision. Fifty percent of patients with multiple sclerosis (MS) experience ON during the course of the disease. In up to 20% of them, ON is the presenting feature of MS [1]. Optical coherence tomography (OCT) allows for non-invasive, reproducible imaging of the live retina and its non-myelinated axons, and serves as a potential biomarker for estimating the neuroaxonal loss in the central nervous system (CNS).

While no animal model can fully recapitulate all pathophysiological aspects involved in MS, some models have proven useful for shedding light on the pathogenic mechanisms underlying neuroinflammatory injury, as well as to test candidate therapeutics. The most commonly studied animal model of MS is murine experimental autoimmune encephalomyelitis (EAE), where mice develop varying degrees of optic neuritis, white matter injury, and ascending myelitis [2].

After immunization of SJL/J mice with PLP_139–151_, Shindler et al. detected loss of retinal ganglion cells (RGCs, the neurons whose axons form the optic nerve) at day 14, starting with a 43% reduction which increased to 50% by day 18. RGC loss was correlated with the severity of inflammation, suggesting that RGC loss in EAE is a direct consequence of optic neuritis [3]. In C57B1/6 mice, active immunization with myelin oligodendrocyte glycoprotein peptide (MOG_35–55_) or adoptive transfer of MOG-specific T cells causes severe optic neuritis [4]. Because MOG expression is higher in the optic nerves than in the spinal cord, even suboptimal doses of MOG can induce experimental optic neuritis in the absence of clinically evident paraparesis in EAE [5]. The TCR^2D2^ mouse line has a MOG-specific T cell receptor leading to severe clinical disability of immunized animals. While TCR^2D2^ mice can develop spontaneous ON more frequently than they develop spontaneous EAE, the use of pertussis toxin (PT) promotes higher, timely incidence of ON and EAE [6]. In this context, Guan et al. found that RGC loss occurred progressively, reaching 39% at day 16 after injection with PT [6]. Horstmann and colleagues studied the retinas and optic nerves of C57B1/6 mice 23 days after the induction of MOG_35–55_ EAE [7]. In this study, the severity of cellular infiltration and demyelination of the optic nerve as assessed by chemical staining techniques (such as hematoxylin-eosin and luxol fast blue staining, respectively) correlated with the clinical EAE score. In this setting, EAE led to an 18% loss of RGC although no change in thickness of any retinal layer could be identified by measuring retinal sections under the microscope likely because of artifacts induced by fixation and tissue preparation.

Although these in vivo models of experimental ON have proven potentially useful to study mechanisms of neurodegeneration and neuronal survival with an autoimmune reaction mediated by stimulating an adaptive immune response, they are mainly focused upon histological measures (either by staining or by retrograde labeling of RGC). Most of these studies had short observational periods of 21 to 40 days after immunization and lacked longitudinal assessments for time tracking of cell injury and loss. Neurodegeneration, however, is a dynamic process. In recent years, the development of non-invasive, retinal imaging techniques for animal models, such as OCT, has helped shed light on the longitudinal neurodegenerative changes during neuroinflammation. This approach has, additionally, emerged as a platform for the preclinical screening of candidate neuroprotective compounds in MS and other neuroinflammatory diseases [8–10].

Studies comparing anterior visual pathway pathology and in vivo imaging characteristics of different EAE models are lacking. A detailed characterization of retinal injury in animals is essential as it can facilitate translational research employing experimental designs that are directly adaptable to human clinical trials. To date, the most appropriate animal model to study ON in EAE has not been defined. The utilization of spectral-domain (SD) OCT in animal models has been constrained by technical and optical issues. To date, although different segmentation algorithms have been tested in volume scans [11], it is not apparent which OCT scanning protocol is most suitable to assess thickness changes of the retinal layers. Acceptable reproducibility and reliability have been reported using a custom-made algorithm for automated segmentation of retinal layers in retinal volume scans from mice [12]. However, in 2D scans, the automatic segmentation of the different retinal layers in mice is often prone to errors. Thus, segmentation of retinal layers is conducted manually or semi-automatically with manual correction, a laborious task that can potentially introduce systematic errors (e.g., due to examiner subjectivity and fatigue bias). It is, therefore, crucial to assess the reproducibility of consecutive assessments and the inter-rater reliability to identify scan protocols associated with high validity and reliability.

This work aimed to describe the dynamics of retinal injury after acute ON in different mouse models of EAE and to define the optimal mouse model, experimental conditions, and analysis methods for the assessment of neuroprotective therapies in EAE using OCT as a primary outcome measure.

## Methods

### Experimental design

As a first step, we investigated which OCT scanning protocols are associated with the best test-retest reliability for retinal layer measurements. We then ran a series of experiments aimed at determining the optimal duration and the ideal mouse model for studying retinal changes in EAE. We analyzed the dynamics of retinal injury after direct immunization against MOG_35–55_ and MBP in C57BL/6J mice, MOG_35–55_ EAE in TCR^2D2^ transgenic mice, and PLP_139–151_ in SJL/J mice. We analyzed changes in retinal thickness as assessed by serial retinal OCT, using sham-immunized, age-matched female mice from the same strain and/or WT littermates for TCR^2D2^ transgenic mice as controls for each model.

### Mice

We bred C57Bl/6J and TCR^2D2^ transgenic mice from parents purchased from the Jackson Laboratories (Bar Harbor, ME, USA). SJL/J mice were provided by S. S. Zamvil, University of California, San Francisco. We performed our experiments on female and male (for TCR^2D2^) 8- to 10-week-old mice.

### Anesthesia

Before imaging, mice were anesthetized by mask inhalation of isoflurane vaporized at concentrations of 1.5% (2 l/min) and their pupils were dilated with 1% tropicamide ophthalmic solution (Akorn, Lake Forest, IL). Compared to the use of intraperitoneal (i.p.) injection of ketamine and xylazine, this approach has proved to be simpler and safer, allowing for rapid induction and easy control of the depth of anesthesia, with a low percentage of complications [13]. Furthermore, this method avoids the xylazine-induced acute, reversible cataract in rodents [14].

### Optical coherence tomography

**Fig. 1:**
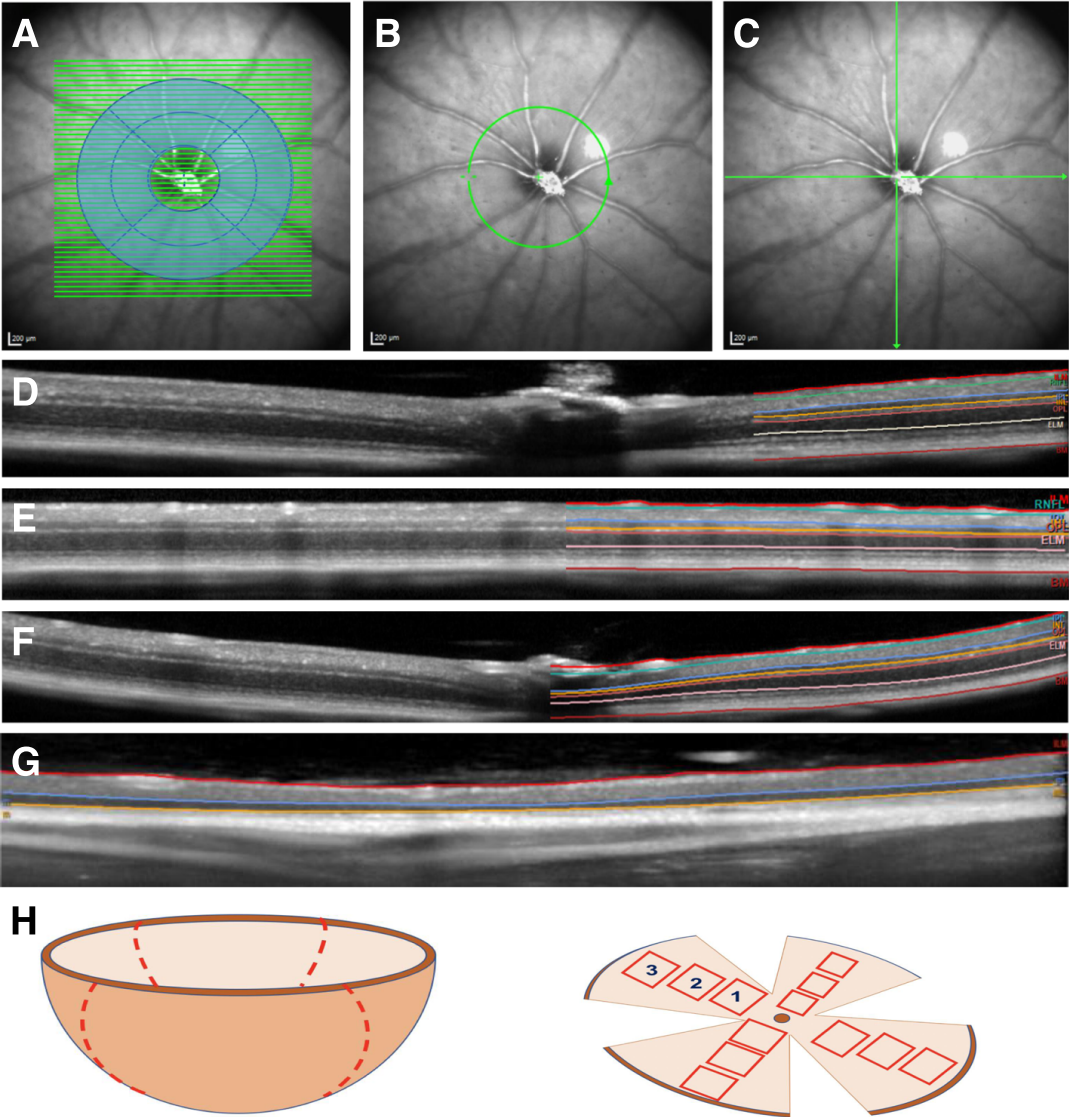
**a**–**c** Fundus image of mouse retina with segmentation of OCT, where each green line depicts a B-scan. **a** Volume scan taken from raster scan of the optic nerve head. Area in blue includes the region of calculated volume. **b** Peripapillary ring and **c** horizontal and vertical line scans. **d**–**f** Examples of B-scans obtained from volume (**d**), peripapillary ring (**e**), and (**f**) line scans. On the right half of each B-scan, the results of the semi-automated segmentation of the layers are shown with retinal layers labeled. **g** Horizontal B-scan in a SJL/J mouse, with atrophy of outer retinal layers, a characteristic finding in retinal degeneration 1. **h** Schematic representation of a retina dissected from an eyeball (left) and a whole mount for histological analysis (right). The dotted line represents cuts made on the retina; the red squares represent areas analyzed in the central (1), mid-peripheral (2), and far-peripheral retina (3)

We performed retinal imaging using Spectralis™ OCT (Heidelberg Engineering, Heidelberg, Germany). During the exams, mice were placed on a custom-made mouse restrainer allowing free rotation and alignment of the eye to ensure the retinal laser was properly centered on the optic nerve head [15]. SD-OCT imaging was performed with and without the help of the TruTrack™ eye tracker that uses the fundus image to achieve imaging, maintaining registration of the image to enhance fidelity brought with averaging and to reduce breathing artifacts. We adapted for the optical properties of the mouse eye by using a custom contact lens during the examination, along with hydroxypropyl methylcellulose 0.3% (GenTeal™ ophthalmic gel, Novartis, Basel, Switzerland) to keep the eye moist and to ensure refraction continuity. Furthermore, we altered the Spectralis™ hardware by adding a 78-diopter lens in front of the camera and by adjusting the length of the reference arm (an option of the Spectralis software). All scans were acquired with an initial focus distance of 42D followed by manual correction. For volume scans, retinal layer thicknesses were calculated using the ETDRS grid with diameters of 1, 2, and 3 mm centered on the optic disc. We calculated the thickness of each retinal layer by averaging each sector of the grid, excluding the center which corresponded to the optic nerve head. Each volume scan consisted of 49 B-Scans recorded in high-resolution mode at 100 ART (rasterized from 100 averaged A-scans). The current version of the software does not allow for the exclusion of the center in horizontal and vertical line scans, which we combined into a crossline by averaging individual horizontal and vertical layer thicknesses. We corrected segmentation artifacts at the disc by drawing straight lines between its opposing borders (Fig. 1). For each experiment, OCT imaging was performed at the time points indicated in the “Results” section.

### Investigation of the test-retest and inter-rater reliability for retinal layer assessments

We performed volume, peripapillary ring, and horizontal and vertical line scans to analyze the thickness of each retinal layer in 10 C57BL/6J mice obtained from Jackson Laboratories (Bar Harbor, ME, USA). We assessed the repeatability of OCT acquisition by removing and replacing the subject mouse onto the mount after each completed scan program, repeating the imaging with and without the Spectralis follow-up function—which utilizes the Spectralis eye tracking function to register images during follow-up scans. We assessed the quality scores (a measure of signal intensity) for each protocol and considered them acceptable if above 20 and excellent if above 30. Using automated segmentation by the Heidelberg Eye ExplorerTM software (version 1.7.1.0 with the 5.10 beta version of the segmentation algorithm) and subsequent manual correction of segmentation errors, we obtained the thickness of the retinal layers on each scan. Two independent investigators determined the segmented thicknesses of each OCT scan. We assessed the total retinal thickness, individual retinal layers, and inner retinal layers (IRLs), defined as the combination of the retinal nerve fiber layer (RNFL) and the ganglion cell and the inner plexiform layer (GCIPL) [16].

### Optokinetic response

The optokinetic response was assessed as a measure for the visual acuity with a testing chamber and the OptoMotry™ software from CerebralMechanics™ (Lethbridge, Canada). A detailed description of the device [17] and methodology [8] is given elsewhere. In brief, we positioned the mice on a platform surrounded by TFT monitors displaying a virtual cylinder of black gratings (100% contrast) rotating in varying directions and at different frequencies. The mice head movements tracking the grating were evaluated by an investigator blinded for the experimental groups. The spatial threshold frequency at which tracking was no longer possible was determined as a measure of visual function.

### Induction and clinical scoring of EAE

#### Direct immunization against MOG_35–55_ in C57Bl/6J and TCR^2D2^ mice

Animals were immunized subcutaneously with 100 μg of MOG_35–55_ peptide (Genemed Synthesis, San Antonio, TX, USA) in complete Freund’s adjuvant (CFA) containing 400 μg *Mycobacterium tuberculosis* (Mt) H37Ra (Difco Laboratories, Detroit, MI, USA). Mice received 200 ng PT (List Biological, Campbell, CA, USA) by i.p. injection at the time of and 48 h post-immunization. Control mice were sham-immunized with phosphate-buffered saline in CFA and received the same PT dosage.

#### Direct immunization against PLP_139–151_ in SJL/J mice

SJL/J mice were injected with 100 μg PLP_139–151_ in 400 μg CFA subcutaneous and 2 × 50 ng PT i.p. on days 0 and 2. Control mice were sham-immunized with phosphate buffer saline in CFA and received the same PT dosage.

#### Direct immunization against MBP in C57Bl/6J mice

Animals were immunized with 400 μg of bovine MBP (Sigma, Darmstadt, Germany), emulsified in 200 μl of CFA, and supplemented with 4 mg of Mt H37Ra, both purchased from Difco. Additionally, mice received i.p. injections of 200 ng of PT (Sigma-Aldrich, Darmstadt, Germany) on days 0 and 2 after immunization. We recorded daily clinical scores, as detailed in Table 1.

**Table 1:** EAE clinical severity scores

0	No signs of disease.
0.5	Mild tail paresis: tip of the tail is weak and/or mouse does not spin tail.
1	Obvious tail paresis or plegia.
1.5	When flipped on its back, the mouse does not turn instantly in > 50% of the cases (this score can only be assigned when signs of tail weakness as described in 0.5 and 1 are present at the same time).
2	Mild signs of hind limb paresis, like abnormal or slow gait, abnormal posture of the posterior part of the body.
2.5	Obvious signs of hind limb paresis, like abnormal, slow, and weak movements of one or both hind limbs.
3	Signs of hind limb plegia: drags one hind limb behind (if the limb is moved a little but it does not help the mouse to move, this will count as a 3).
3.5	Signs of hind limb plegia: drags both hind limbs behind (if the limbs are moved a little but it does not help the mouse to move, this will count as a 3.5).
4	Mild signs of quadriparesis (weakness of all 4 limbs), as described in 2–3.5 and signs of weakness of one or both front limbs, like reduced speed when pulling itself forward, inability to push its chest up from ground, or reduced ability (shorter duration) to hold itself up against gravity on the edge of the cage.
4.5	Quadriplegia: cannot or barely pull itself forward or hold itself on the edge of the cage (in this stage the mouse has to be monitored closely and has to be sacrificed before 24 h if the condition does not improve).
5	Mouse found dead.

### Post-acquisition analysis

Using automated segmentation by the Heidelberg Eye Explorer™ software (version 1.7.1.0 with the 5.10 beta version of the segmentation algorithm) and subsequent manual correction of segmentation errors, we obtained the thickness of the retinal layers on each scan. For volume scans, retinal layer thicknesses were calculated using the early treatment of diabetic retinopathy (ETDRS) study grid (1, 2, 3 mm) centered on the optic disc (Fig. 1). We calculated the thickness of each retinal layer by averaging each sector of the grid, excluding the center which corresponded to the optic nerve head. In line scans, we corrected segmentation artifacts at the disc by drawing straight lines between the opposing borders of the optic disc. We determined the thickness of individual retinal layers and the IRL [16]. Thickness data were exported from the segmentation software onto an Excel spreadsheet (Microsoft, WA, USA). In the case of SJL/J mice, which are homozygous for the allele Pde6b^rd1^ (retinal degeneration 1), we used an 8 × 8 grid, obtaining retinal thickness values only in those sectors where imaging of the inner retinal layers was feasible (Fig. 1d).

### Histological analysis and immunofluorescence microscopy

Mice were sacrificed with an overdose of isoflurane. Cardiac perfusion was performed, and optic nerves and retinae were dissected. Optic nerves were fixated in 4% paraformaldehyde (PFA) overnight and dehydrated in sucrose solutions with increasing concentrations. After embedding in OCT compound (Sakura™ Finetek), longitudinal sections of 5 μm were cut for immunohistological analysis. The antibodies used for immunofluorescence microscopy are listed in Table 2. To examine adaptive immune infiltration by CD3+ lymphocytes and innate immune activation—microglial/macrophage infiltration—as well as the myelin status of the optic nerves, slices were incubated with CD3 (1:400, Dako), Iba1 (1:500, Wako chemicals), and MBP (1:500, Millipore) antibodies, respectively. For a further rating of immune cell infiltration in optic nerves, hematoxylin and eosin (HE) staining was performed. RGC count was calculated by a semi-automated count of βIII-tubulin- or Brn3a-positive cells on retinal flatmounts. Briefly, retinae were stained with Brn3a (1:200, Santa Cruz Biotechnology, cat # sc-31984) or βIII-tubulin antibody (1:1000, Biolegend) and flat-mounted on glass slides. Each retina was then divided into four quadrants (three areas per quadrant: central, mid-periphery, and far-periphery) (Fig. 1e). For each eye, Brn3a+ or βIII-tubulin+ cell count was summed up from all 12 areas imaged as previously described [18].

**Table 2:** List of antibodies used for immunofluorescence microscopy

	Type	Manufacturer	Catalog #	Host species	Dilution used
Primary antibodies	Anti-CD3	Dako, Hamburg, Germany	A0452	Mouse	1:400
	Anti-Iba1	Wako, Richmond, VA, USA	019-19741	Rabbit	1:500
	Anti-MBP	Millipore, Burlington, MA, USA	MAB386	Rat	1:500
	Anti-Brn3a	Santa Cruz Biotechnology, Dallas, TX, USA	sc-31984	Goat	1:200
	Anti-βIII-tubulin	Biolegend, San Diego, CA, USA	801201	Mouse	1:1000
Seconday antibodies	Alexa Fluor-555 Anti-Goat	Life Technologies, Carlsbad, CA, USA	A-21432	Donkey	1:200
	Alexa Fluor-488 Anti-Rabbit	Life Technologies, Carlsbad, CA, USA	A-21206	Donkey	1:200
	Cy3 anti-Rabbit	Millipore, Burlington, MA, USA	AP187c	Goat	1:500
	Cy3 anti-Mouse	Millipore, Burlington, MA, USA	AP124c	Goat	1:500
	Cy3 anti-Rat	Millipore, Burlington, MA, USA	AP183C	Goat	1:500

### Electron microscopy

For transmission electron microscopy (TEM), mice were sacrificed and cardiac perfusion was performed with 2% PFA and 2.5% glutaraldehyde (GA). Optic nerves were dissected and incubated in the fixative containing 2% PFA and 2.5% GA at 4 °C for 3 h, followed by incubation in 1% osmium tetroxide for 2 h. Dehydration was achieved using acetone at increasing concentrations, and block contrast was applied (1% phosphotungstic acid/0.5% uranylacetate in 70% acetone). A Spurr embedding kit (Serva, Heidelberg, Germany) was used according to the manufacturer’s protocol. Ultrathin sections of 70 nm were cut using an Ultracut EM UC7 (Leica) and stained with lead-citrate [19] and 1.5% uranyl-acetate. Images were captured at various magnifications using a TEM H7100/100KV (Hitachi, Tokyo, Japan) using a Moroda SIS Camera system and were subsequently processed by Olympus ITEM 5.0 Software.

### Statistical analysis

We performed the statistical analysis with SPSS version 22 (IBM). Data are presented as mean ± standard error of the mean (SEM). We calculated the area under the curve of EAE daily scores for each group. For all OCT scans, we calculated the two-way mixed effect absolute agreement interclass correlation coefficient (ICC) to assess the reliability of the measurements of every layer obtained by the two independent raters (“inter-rater reliability”) and to assess the repeatability of consecutive scans (“test-retest reliability”). We report the ICC and the 95% confidence intervals (CI). ICC values above 0.9 were considered as excellent, between 0.8 and 0.9 as good, between 0.7 and 0.8 as acceptable, between 0.6 and 0.7 questionable, between 0.5 and 0.6 as poor, and below 0.5 as unacceptable [20]. Differences in retinal thickness were analyzed using generalized estimating equations (GEE) with an exchangeable correlation matrix to adjust for intrasubject inter-eye correlations. We studied the association of OCT results with EAE severity and RGC loss by GEE association analyses, to identify which measurements could be used as surrogates for neuronal injury. Differences in RGC survival were analyzed with a one-way ANOVA and Holm-Sidak post hoc test. *P* values are designated as follows: *P ≤ 0.05, **P ≤ 0.01, and ***P ≤ 0.001. For calculation of the sample sizes required for neuroprotection studies, we performed a power analysis using G*Power (Version 3.1.9.2) for an α of 0.05 and a power (1-ß) of 0.8.

## Results

The OCT quality scores were good for the ring and excellent for cross and volume scans (Additional file 1: Figure S1).

### Inter-rater reliability

Semi-automated segmentation of the IRL was feasible for all retinal layers in all three scanning protocols (Fig. 1). Volume scans provided excellent inter-rater reliability with ICC values of above 0.9 for all assessments (Additional file 2: Figure S2). Cross scans (average of horizontal and vertical line scans) and peripapillary ring scans provided a good to excellent inter-rater reliability for the assessment of the IRL thickness (ICC 0.961, 95% CI 0.913–0.983, and ICC 0.816, 95% CI 0.468–0.932, respectively) while the RNFL showed good reliability for cross scans and unacceptable reliability for ring scans (ICC 0.828, 95% CI 0.533–0.930, and ICC 0.278, 95% CI − 0.244–0.666, respectively).

### Test-retest reliability

We obtained the highest reproducibility with volume scans, which showed excellent reliability for the assessment of the IRL and the GCIPL (Additional file 3: Figure S3). Cross and peripapillary scans provided excellent reproducibility only for the measurement of IRL thickness (0.918 [95% CI 0.492–0.983] and 0.950 [95% CI 0.508–0.993]) and, in the case of cross scans, also for the GCIPL (0.937 [95% CI 0.746–0.985]) while the RNFL showed a poor reliability for cross scans and a questionable reliability for ring scans (0.551 [95% CI − 0.568–0.89] and 0.624 [95% CI − 0.655–0.937]). The follow-up function did not add significantly to reproducibility.

In summary, in vivo retinal imaging by OCT was associated for a high inter-rater and test-retest reliability for the IRL and can, therefore, be used for the in vivo monitoring of neuroaxonal loss, e.g., in the context of optic neuritis.

A summary of the longitudinal experiments performed in EAE and their results is outlined in Table 3.

**Table 3:** Summary of EAE experiments and results

Figures	Strain	Encephalytogenic peptide	OCT follow-up period	OCT time points (months after immunization)	OCT findings	Histological findings	Additional findings
2, 3, 4, 6	WT C57Bl/6J	MOG_35–55_	120 days	0, 0.5, 1, 2, 3, 4	Initial IRL swelling mirroring EAE onset and peak, followed by progressive thinning	Optic neuritis (T cell infiltration, microglial activation, and demyelination) with 32% RGC loss. Decrease of myelinated nerve fibers and destruction of the myelin structure.	Decreased visual acuity (OKR)
2, 3	WT C57Bl/6J	MBP	120 days	0, 0.5, 1, 2, 3, 4	No change	No optic neuritis or RGC loss	
2, 3	TCR^2D2^	MOG_35–55_	120 days	0, 0.5, 1, 2, 3, 4	IRL atrophy already present at 2 weeks	Severe optic neuritis with 49% RGC loss	
5	WT C57Bl/6J	MOG_35–55_	9 months	0, 1, 2, 5, 7, 9	Progressive IRL and INL thinning, more pronounced during the first 2 months	54% RGC loss	
6	SJL/J	PLP_139–151_	7 months	0, 1, 2, 5, 7	Progressive IRL atrophy, also present to a lesser degree in sham-immunized mice		

### Retinal changes during 120 days after immunization with MOG_35–55_ or MBP in WT C57Bl/6J and with MOG_35–55_ in TCR^2D2^ mice

IRL swelling was evident 2 weeks after immunization of WT mice with MOG_35–55_, which corresponded to clinical EAE, and was followed by progressive thinning and loss of RGC.

**Fig. 2:**
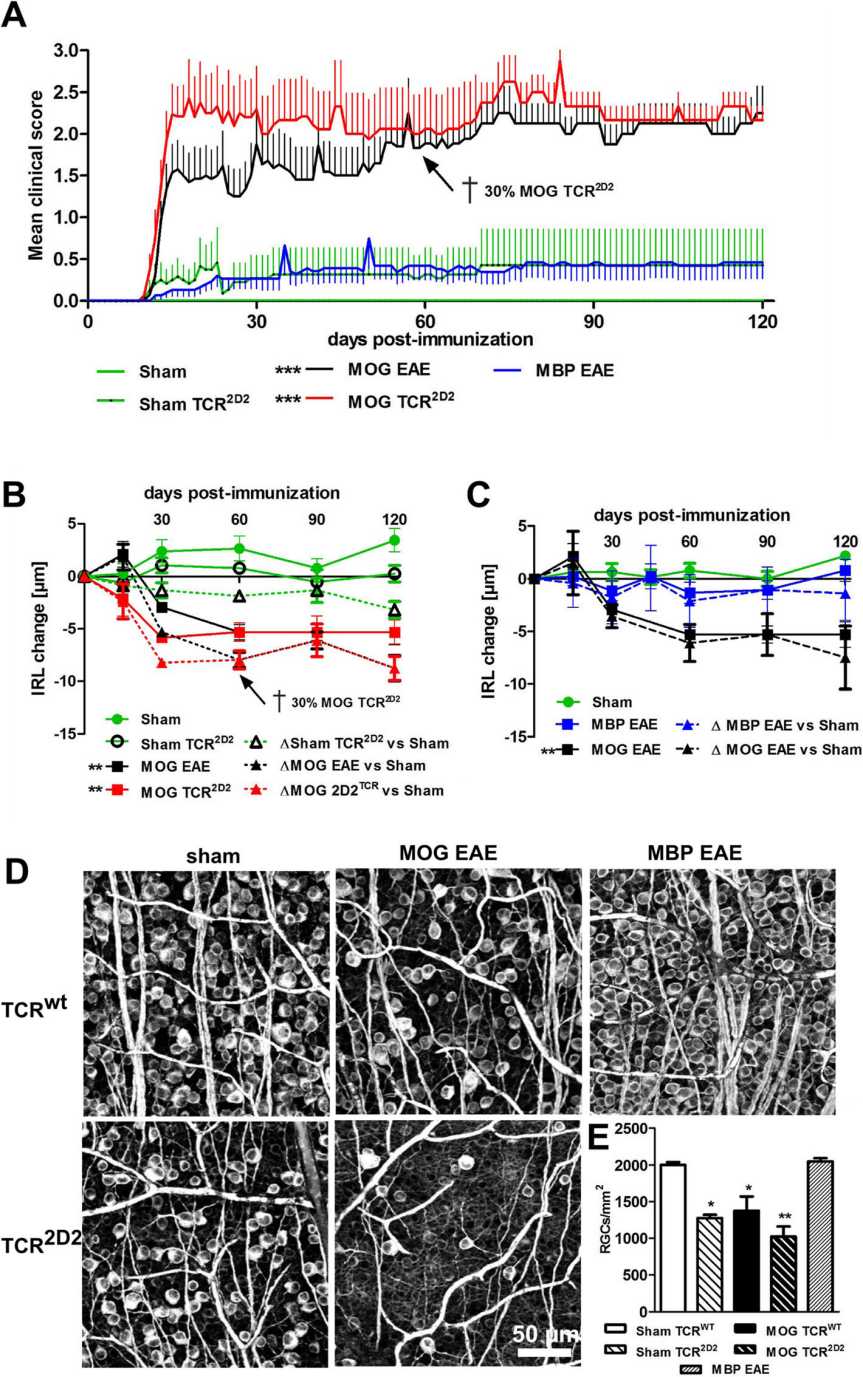
**a** Clinical EAE score of C57Bl/6J mice immunized with MOG or MBP peptide, as well as TCR^2D2^ transgenic mice immunized with MOG peptide. **b** Change of the inner retinal layers of TCR^WT^ and TCR^2D2^ mice. **c** IRL change of C57BL/6J mice immunized with MBP. **d** Retinal wholemounts stained by βIII-tubulin antibody from groups indicated in **a**. The representative images display the most severe disease progression. **e** The bar graph shows the RGC density 120 days after immunization. Time courses and bar graphs represent the pooled mean ± SEM of two separate EAE experiments each with at least four animals per group, *P > 0.05; **P < 0.01; ***P < 0.001; time courses (area under the curve) were compared to sham, and bar graph compared to sham TCR^WT^ mice by ANOVA with Dunnett’s post hoc test

After MOG_35–55_ immunization, TCR^2D2^ mice developed a severe clinical disability. Those suffering from quadriparesis had to be fed with liquid gel and soaked chow. Some animals (*n* = 2), that received sham immunization (PT and CFA), developed spontaneous clinical signs. Although C57BL/6 mice are often considered resistant to EAE induced by MBP, some data suggest otherwise [21, 22]. Thus, we studied the disease course and phenotype of retinal injury in this model as well. Clinical signs were minimal and started around the same day as MOG_35–55_-induced EAE (Fig. 2).

Contrary to our observations in WT C57Bl/6J mice immunized with MOG_35–55_, IRL thinning was already evident after 2 weeks in TCR^2D2^ mice. However, after 60 days, no differences were observed between WT C57Bl/6J and TCR^2D2^ mice anymore, mainly because TCR^2D2^ animals (~ 30%) were sacrificed due to development of severe clinical EAE, reducing the number of animals with strong IRL degeneration. Mean IRL of the sham TCR^2D2^ group showed no thickness increase after a period of 120 days, while healthy WT controls gained up to 4 μm compared to the baseline measurement, suggesting that ON neurodegeneration occurs in TCR^2D2^ mice also in the absence of clinical EAE signs (Fig. 2b). As IRL thickness in control mice was subject to dynamic changes, we also analyzed the difference between EAE mice and the average thickness of healthy control mice, at each time point after immunization. This allowed for better visualization of the pace and extent of retinal atrophy after demyelinating injury in EAE mice. In line with the clinical disability of MBP-immunized mice, neither degeneration of the IRL (Fig. 2c) nor the total retina (data not shown) was detected by OCT analysis and no significant reduction of retinal ganglion cells (RGC) (Fig. 2d, e) was observed at 120 days after MBP immunization compared to the sham control. Staining of the RGC from sham TCR^2D2^ mice confirmed the data of the OCT measurements, showing a reduced number of βIII-tubulin-positive cells in the retinae, also in those with no limb paresis. MOG-immunized TCR^2D2^ mice showed strong RGC reduction but also high variances between the single individuals (Fig. 2d, e).

**Fig. 3:**
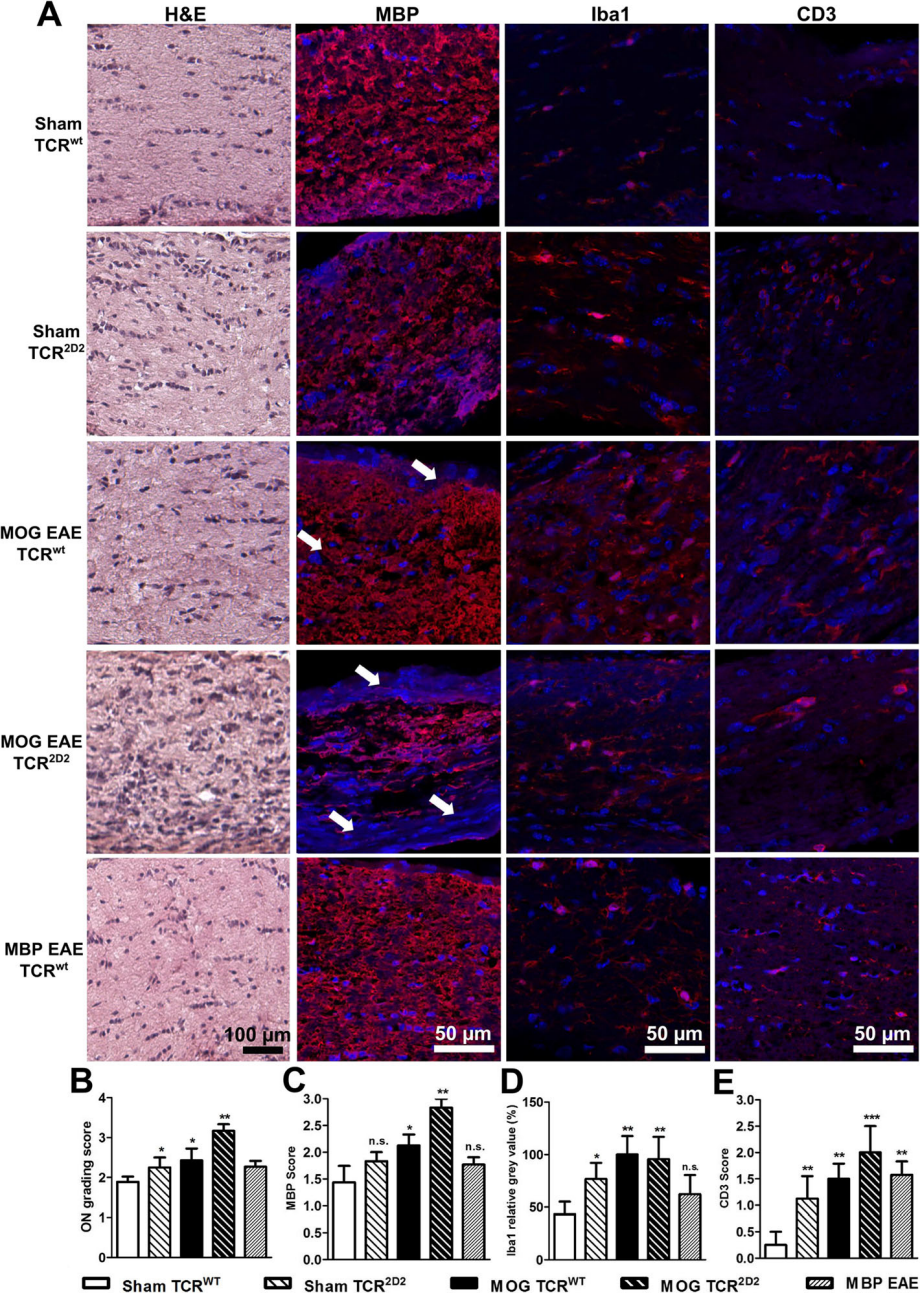
**a** Optic nerves of mice 120 days after MOG or MBP immunization stained by H&E and CD3, Iba1, or MBP antibodies. Optic nerves were compared by an established infiltration score according to H&E staining (**b**) (Shindler et al. [3]), MBP score for the myelin status (**c**), Iba1 fluorescence intensity measurement for microglia activation (**d**), and CD3+ cell infiltration (**e**). The bar graphs represent the pooled mean ± standard deviation of at least two separate EAE experiments each with at least four animals per group; one optic nerve per mouse was included, *P < 0.05; **P < 0.01; ***P < 0.001; n.s., not significant, by ANOVA with Dunnett’s post hoc test compared to sham-treated mice

HE-stained optic nerves from sham-immunized TCR^2D2^ mice showed immune cell infiltration, compared to sham WT littermates, demonstrating the presence of ON, also in animals with no clinical signs. TCR^2D2^ mice immunized with MOG_35–55_ had severe inflammatory infiltration, exceeding the ON score of the WT control. Staining of the optic nerve revealed no significant cumulative infiltration of immune cells after MBP immunization, suggesting a mild disease pathology (Fig. 3).

Increased T cell infiltration, microglial activation, and demyelination of the optic nerve were observed 120 days after immunization of WT mice with MOG_35–55_ peptide. Microglia activation, as well as T cell infiltration, was even present in TCR^2D2^ mice after sham immunization with CFA and PT. Even more severe infiltration of CD3-positive T cells into the optic nerve and myelin degradation was observed in TCR^2D2^ EAE mice compared to WT animals, while microglial activation was similar (Fig. 3). Despite no significant changes in cellular infiltrates assessed by HE staining, we observed an increased infiltration of CD3-stained T cells at day 120 in optic nerves of MBP-immunized mice, while microglia activation and demyelination was unaltered.

**Fig. 4:**
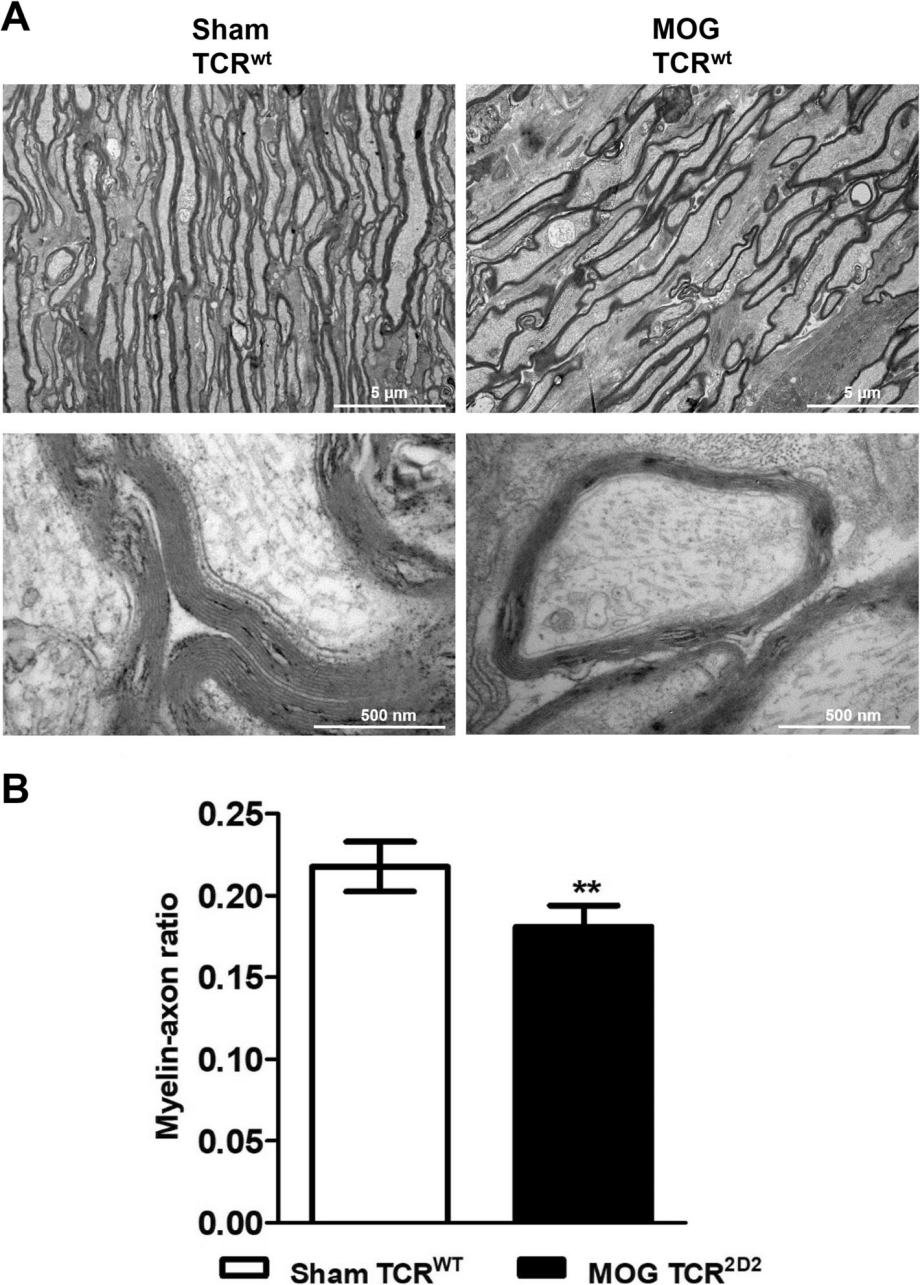
**a** Electron microscopy images of longitudinal sections of the optic nerve fibers from sham- and MOG_35–55_-treated C57Bl/6J wild-type mice. **b** The myelin-axon ratio was determined by the thickness of the myelin sheath and the axon. Bar graphs represent the pooled mean ± SD of an EAE experiment with four animals per group; **P < 0.01 two-tailed Student’s *t* test compared to sham-treated mice

To determine the effects of EAE on the ultrastructure of the myelin sheath, we performed electron microscopy of ultrathin cross sections of the optic nerve from sham- and MOG_35–55_-immunized C57Bl/6J mice. Macroscopic analyses of the optic nerves revealed a normal myelin sheath in the sham-immunized group while optic nerves of EAE mice displayed a prominent decrease of myelinated axons and destruction of the myelin structure (Fig. 4a). A quantitative analysis of the myelin structure revealed a significantly lower myelin-axon ratio in MOG- compared to sham-immunized mice confirming the impression of myelin damage in MOG EAE from our macroscopic investigations (Fig. 4b).

In summary, immunization of C5Bl/6J mice with MOG_35–55_ peptide but not MBP induced robust EAEON, detectable by ON histology and leading to a degeneration of the IRL and RGCs detectable by OCT and histology, respectively. This was even more pronounced and often leading to death in TCR^2D2^ mice, which also displayed mild symptoms of ON when sham-immunized despite no motor symptoms.

### Chronic retinal changes during 9 months after immunization and visual testing in MOG EAE in WT C57Bl/6J mice

**Fig. 5:**
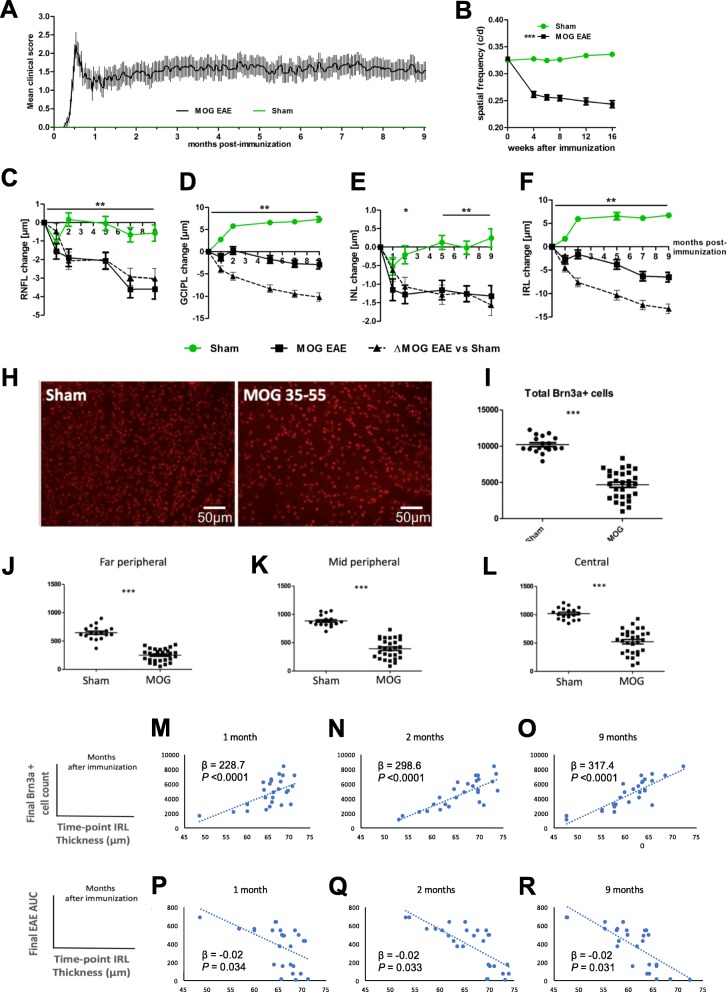
MOG_35–55_ EAE in C57Bl/6J (*n* = 17) vs. sham-immunized (*n* = 12) mice. **a** EAE clinical scores. **b** Decreased visual acuity of EAE mice compared to untreated C57Bl/6J mice. OKR measurement was carried out for 120 days after MOG immunization as described above, area under the curve compared by ANOVA with Dunnett’s post hoc test. **c**–**f** Thickness of retinal layers. **h**–**l** RGC count after 9 months. Data expressed as mean ± SEM, *P < 0.05; **P < 0.01; ***P < 0.001. *P* values for OCT and RGC data obtained from generalized estimating equation models accounting for within-mouse, inter-eye correlations. **m**–**r** Linear regression analyses. IRL thickness during EAE is associated with ultimate neuronal loss (top row) and disease severity (bottom row). Similar results were obtained 5 and 7 months after immunization (data not shown). ß, generalized estimating equation association coefficient

In the eyes of control animals, we observed a progressive thickening of the retinae mirroring weight gain most likely corresponding to the natural growth of the eyes of the 6–8-week-old mice (Fig. 5a). In EAE eyes, progressive thinning of the IRL started rapidly after the disease onset, with 1/3 of total loss occurring during the initial 2 months (− 4.25 ± 0.87 μm compared to controls, *P* < 0.001 at month 2; − 13.39 ± 1.33 μm, *P* < 0.001 at month 9). Inner nuclear layer (INL) thinning was detectable from the second month after immunization in EAE mice while INL thickness remained constant throughout the experiment in control mice.

As we identified the WT C57Bl/6J mice as a very suitable model for studying retinal injury in EAE, we chose to investigate the functional outcome of the structural results. Visual function was assessed by the optokinetic response of the mice, using the spatial frequency threshold as a readout for visual acuity. It was significantly reduced in EAE mice with values of 0.23 cycles per degree (c/d) compared to 0.33 c/d for sham-EAE control mice after 120 days of EAE (Fig. 5c).

After 9 months, the RGC density halved (4665.83 ± 360 total Brn3a cells counted per eye versus 10,206.78 ± 265, *P* < 0.001) in immunized mice. This finding was consistent along all three retinal sectors (central, mid-peripheral, and far peripheral) (Fig. 5d).

Investigating the IRL thickness at each time point (1, 2, 5, 7, and 9 months after immunization), we analyzed its association with the final RGC count (obtained at the end of the experiments, 9 months after immunization) and the total EAE score area under the curve (an indicator of the overall clinical severity and burden of disease) (Fig. 5e). IRL thinning during EAE was significantly associated with RGC loss (ß GEE association coefficient 317.4, 95% CI 241.1, 393.7, *P* < 0.0001 at month 9 after immunization) and disease severity (ß − 0.02, 95% CI − 0.03, − 0.001, *P* = 0.031 at month 9).

### Chronic retinal changes during 7 months after immunization in PLP_139–151_ EAE in SJL/J mice

**Fig. 6:**
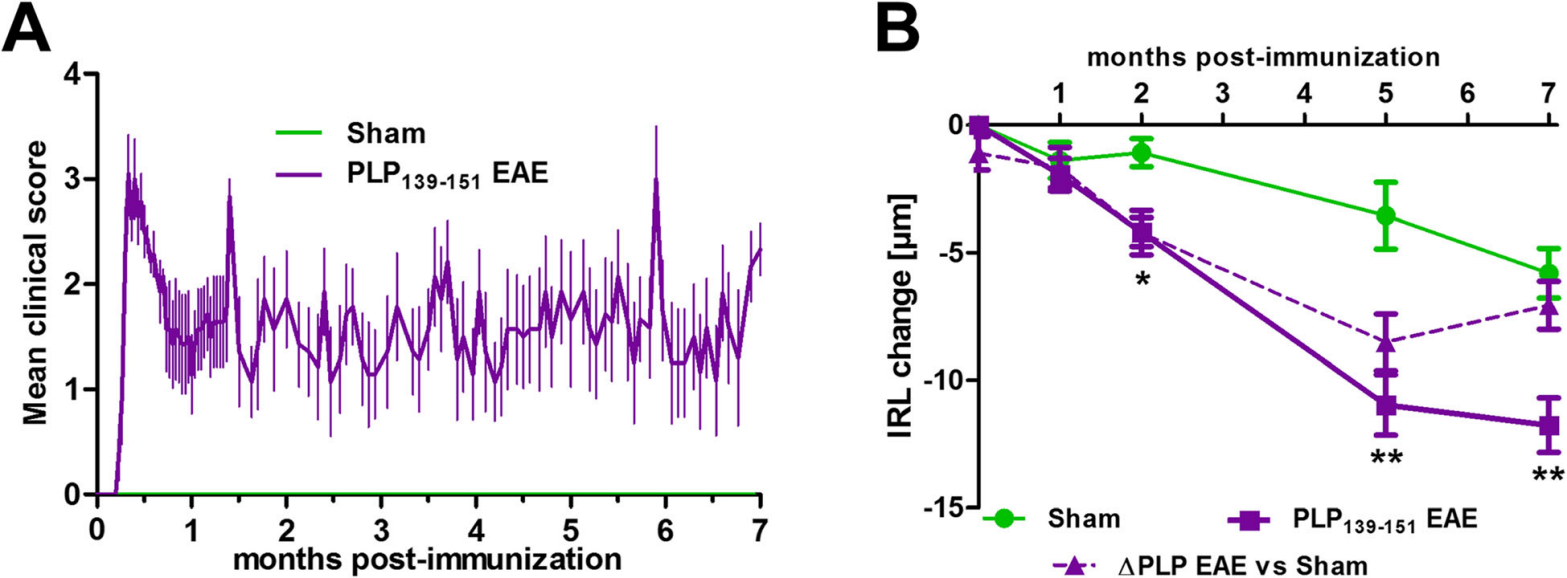
**a** Clinical EAE scores in PLP_139–151_ EAE in SJL mice. The abscissa axis represents days after immunization. **b** IRL thickness in PLP_139–151_ EAE (*n* = 10) vs. sham-immunized (*n* = 5) SJL/J mice. Data expressed as mean ± SEM; *P < 0.05; **P < 0.01

We sought to characterize the dynamic changes in retinal thickness in SJL/J mice following immunization against PLP_139–151_. In this mouse strain, not only immunized mice but also sham-injected controls showed progressive IRL atrophy, due to the retinal degeneration linked to the homozygous mutation of the Pde6b^rd1^ gene. However, in PLP-immunized mice, the IRL thinning was significantly aggravated after the second month following immunization (Fig. 6).

We concluded that the retinal degeneration also occurring in sham-injected controls makes the SJL mouse strain suboptimal for studies investigating the effects of optic neuritis.

### Power analysis for neuroprotection studies

The results outlined above suggested that MOG_35–55_ EAE in C57Bl/6J model is particularly well suited for the investigation of therapeutic strategies aiming at preventing neuroaxonal loss. In this context, it is important to know the number of mice necessary to detect efficacy for given effect size. To this end, we used data from a dataset from our recently published work on the protective effects of alpha-lipoic acid in EAE (Dietrich et al. [8], *J Neuroinflammation*) comparing the results of the different structural and functional readouts in a power analysis. For OCT measurements at the endpoint 120 days after immunization, we determined a minimum sample size of 14 animals per group to detect the 20% difference in IRL thickness change observed at an effect strength of 1.1, considering that the mean of the inner retinal layers changed by 1.7 μm in vehicle-treated mice and was associated with a standard deviation of 1.52 μm. By performing several longitudinal measurements for each animal, the sensitivity to detect treatment effects can be further increased. As a functional measure, a sample size of 3 animals per group was determined to detect alteration of the visual acuity of 15% at an effect strength of 3.7 (0.2286 c/d ± 0.011 c/d to 0.2686 c/d ± 0.011 c/d). For histological staining of RGCs in retinal wholemounts and the clinical EAE score at 120 days, the power analysis determined a sample size of 3 and 18 animals per group, respectively, to detect a change as low as 25% RGC survival and 50% EAE score at an effect strength of 4.45 (1181 RGCs/mm^2^ ± 200 RGCs to 1535 RGCs/mm^2^ ± 200 RGCs) and 0.93 (2.26 EAE score ± 1.27 vs 1.19 EAE score ± 0.93), respectively.

In summary, based on the results of our previous study, we can conclude that OCT and OKR in the EAEON can detect protective treatment effects of 20 and 15% on retinal structure and visual function, respectively, with reasonable sample sizes.

## Discussion

Retinal OCT is increasingly used as an outcome measure for clinical trials of candidate neuroprotective drugs in acute ON [23–26] and MS [27, 28]. Preclinical investigations in experimental ON using in vivo retinal outcomes can be readily transferred to a clinical trial scenario, yet the models best suited to address the different aspects of retinal neurodegeneration by in vivo OCT are still unknown. For this purpose, establishing the dynamics of retinal neuroaxonal loss in EAE is also crucial. Traditionally, EAE experiments mostly focus on clinical and/or purely immunological aspects and extend for 1 month. Our results indicate that in the MOG_35–55_ EAE model in C57BL/6J mice, IRL thickness changes during the course of the disease, reflecting the clinical severity and RGC survival. Here, we observed that slow retinal thinning after peak EAE continues until month 7 even though the clinical signs remain stable. EAE scores almost exclusively represent changes in motor function. This mirrors the current situation in the clinic and in clinical trials, where disability is measured through the EDSS scale, which relies heavily on the ambulatory capacity of patients. Additionally, while EAE scores and EDSS are ordinal scales and depend on the subjectivity of the scorer, OCT provides an objective, continuous quantitative anatomical readout.

We first analyzed the reproducibility and reliability of retinal OCT measurements in mice. Altogether, volume scans offered the best results, while the poorest were obtained with the peripapillary ring scans which, in fact, have been studied as an outcome parameter in animal models of MS [29]. A possible explanation is that the variability of the segmentation of single B-scans is averaged out when analyzing the mean of the 49 scans making up the volume scan. Our data indicate that in mice, the separate assessment of the RNFL and GCIPL thickness can be challenging, since the segmentation of the limit between them is not very reproducible. We, therefore, also analyzed them jointly as the IRL, which yielded much more robust results. Another advantage of evaluating these layers together is that it reflects a combined outcome parameter for axonal (RNFL) and neuronal (GCIPL) loss.

We then investigated the extent and dynamics of retinal injury in different mouse EAE models. The genetic background of a mouse line, as well as the epitope used for immunization, affects the immune and inflammatory response during EAE. In Swiss-derived strains (e.g., SJL/J), PLP immunization leads to a relapsing remitting disease course, while mice with a Bl/6 background develop a more chronic progression, thereby addressing different forms of the inflammatory CNS disease [30]. Additionally, for neuroprotection studies, it may be particularly relevant to use models displaying severe retinal damage to ensure that even small effect sizes of neuroprotective interventions are detectable through OCT. We, therefore, chose to also include the model of PLP_139–151_-induced EAE in SJL/J mice and MOG_35–55_-induced EAE in TCR^2D2^ mice as models with particularly severe RGC degeneration [3] and ON, besides MOG_35–55-_induced EAE in C57BL/6 WT mice [5]. Furthermore, we aimed to investigate the retinal degeneration in MBP-induced EAE in C57Bl/6J mice as a model characterized by a monophasic disease course and predominantly axonal damage but less demyelination [31]. In general, EAE induction in C57Bl/6J mice by MBP is not very feasible [21, 22, 32]; nevertheless, we were able to induce mild EAE signs with whole rat MBP, yet with no significant retinal thinning or relevant pathology of the optic nerve. Hence, we conclude that this model is not suitable for retinal studies. EAE in SJL/J mice following immunization against PLP_139–151_ could be used to study retinal neurodegeneration in the context of repeated relapses. Even though OCT detected changes attributable to EAE in the retinas of SJL/J mice, the concomitant retinal dystrophy hinders further neurophysiological and histological investigations and significantly limits the usefulness of this mouse strain for our purposes. We, therefore, focused on mice with the C57BL/6J background, obtaining results by the findings of Knier et al. [9] and our own previous work [8, 33]. We sought to also characterize the changes in retinal thickness during the acute phase of the disease. Serial OCT 6, 15, 30, 60, 90, and 120 days after immunization revealed three distinctive phases of retinal injury: no changes in retinal thickness were detectable 6 days after immunization (pre-EAE onset, data not shown); coinciding with clinical onset and peak of disease, there was considerable swelling of the IRL in WT mice, while retinal degeneration in TCR^2D2^ mice stared already at this early time point. The chronic phase of EAE was characterized by progressive IRL thinning below baseline levels both in WT and TCR^2D2^ mice. Histopathological examination revealed ON with T cell and microglial infiltrates leading to RGC loss in the absence of clinical signs in TCR^2D2^ mice injected only with PT, in line with the observations of Guan et al. [17]. As IRL thickness is only slightly decreased at day 30 compared to baseline, loss of RGC might not be evident only 1 month after immunization. Taken together, these analyses indicated that in the MOG_35–55_ EAE model through direct immunization of C57BL/6J mice, experiments using retinal OCT as a surrogate for neuronal damage should last more than 1 month and use IRL thickness as their primary outcome.

We measured retinal thickness until changes relative both to baseline levels and age-matched controls stabilized, which happened around month 9 after immunization. This continuation of IRL thinning far beyond the clinical stabilization of EAE suggests that OCT might be more sensitive to assess the chronic neurodegeneration after an acute inflammatory insult. Although significant IRL atrophy was already detectable after 1 month, these changes corresponded exclusively to the GCIPL, and only from the second month were we able also to detect thinning in the RNFL. By the end of the experiment, however, relative thinning of the RNFL was higher than that of the GCIPL. This aligns with studies of ON in humans describing GCL thinning as an early feature [34], detectable before that of the peripapillary RNFL [35], but with ultimate macular RNFL atrophy being more extensive than that of the macular GCIPL [36]. The different timing of RNFL versus GCIPL atrophy has been related to early edema of the RNFL during acute ON or to RGC shrinkage and loss before axonal atrophy occurs. According to this interpretation, RGC degeneration in ON would be driven by two related mechanisms: an initial wave of RGC injury, caused by early signals from damaged axons in the optic nerve, and a later wave of RGC loss, as a consequence of a dying-back process following axonal loss [36]. Our findings in the animal laboratory seem to support these observations. Like in patients with ON [37], we saw changes in the IRL thickness occurring most rapidly in the first months after the acute episode.

Nine months after immunization, surviving RGC were halved in MOG- versus sham-immunized mice. However, measuring IRL thickness longer than 2 months did not show to improve the association of IRL thickness and RGC survival substantially. These results indicated that MOG_35–55_ EAE studies using OCT as a surrogate for the ultimate survival of RGC do not necessarily provide additional meaningful information beyond 2 months after direct immunization unless investigating mechanisms of repair or specifically targeting processes that occur in the late phase. Similarly, because in the MOG-EAE model there is little clinical change beyond the fourth week after immunization, IRL thickness measured at 2 months was as good of a predictor of overall burden of disease (cumulative EAE score) as that measured 1 or 9 months after immunization (*r*^2^ 0.51, 0.47, and 0.52 at 1, 2, and 9 months, respectively).

It should be noted that OCT is a method to image tissue. It lacks real cellular resolution. Therefore, averaging while enhancing reproducibility does not necessarily improve fidelity. Each scan is the result of averaging 30 to 100 images, so the details of the interface between retinal layers might not be fully detected. We did not compare the results with the retinal layer thickness measurements to the layer thickness in histological sections or assessments by other OCT devices or segmentation algorithms. However, this was done in other studies [11, 38] and was not the focus of our work. As a result of these limitations, measurements are obtained from an idealized representation of the retina and can be influenced by pathological changes that impact the optical properties of the tissue under study.

The power analysis using our previously published results with alpha-lipoic acid [8] revealed that protective effects of 20% on the retinal structure could be detected with 14 animals per group, which is in line with other studies using similar methodology [9, 39, 40]. Interestingly, OKR analysis of visual function and RGC counting in retinal wholemounts showed less variance resulting in numbers as low as 3 animals per group in the power analysis to detect 15% protection of visual function and 25% protection of RGCs. Of course, the power achieved by the different modalities depends on the mode of action of the therapeutic approaches studied.

## Conclusions

IRL thickness, as assessed by retinal OCT, is a good surrogate for clinical severity and neuronal loss and survival in mouse models of EAE. During the onset and peak of disease, there is acute IRL thickening, followed by progressive thinning. In MOG-EAE in C57BL/6J mice, this occurs most rapidly between the height of disease and the 60th day after immunization, and only from that time point on is it possible to detect associated thinning of the INL. In the otherwise healthy SJL/J adult mouse, which is a homozygous carrier of the allele Pde6b^rd1^, there is progressive IRL thinning due to retinal dystrophy which is aggravated upon immunization with PLP_139–151_. No significant retinal changes are found in MBP-EAE in C57BL/6J mice. MOG immunization in TCR^2D2^ mice results in severe EAE, therefore challenging animal care and survival during experiments.

We conclude that among the models tested, MOG_35–55_-induced EAE in C57Bl/6J is the most convenient to study retinal neurodegeneration in the context of ON.

## Supplementary information

**Additional file 1: Figure S1.** Quality scores (a measure of signal intensity). Quality above 20 is considered acceptable, quality above 30 is considered excellent.

**Additional file 2: Figure S2.** A: OCT scans segmented by two independent raters with results plotted along a linear regression line. Each point represents a single eye of a mouse. The dotted line the reference for 100% agreement between both raters. Note the relatively improved performance characteristics for volume scans over line scans. B: Interclass correlation coefficients in the different protocols analyzed.

**Additional file 3: Figure S3.** Serial OCT scans of wild-type mice segmented by a single rater. Note: animals were entirely removed and repositioned between scans. Table demonstrates the interclass correlation for different scan protocols. Note volume scans outperformed line scans and follow-up function adds little benefit to reproducibility. In addition, aggregating layers into either total retinal thickness or inner retinal layers generally outperforms individual layers.

## Data Availability

The datasets used and/or analyzed during the current study are available from the corresponding author on reasonable request.

## Abbreviations

ARVO: Association for Research in Vision and Ophthalmology
AUC: Area under the curve
CI: confidence interval
CFA: Complete Freund’s adjuvant
CNS: Central nervous system
EAE: Experimental autoimmune encephalomyelitis
GCIPL: Ganglion cell-inner plexiform layer
GEE: Generalized estimating equations
HE: Hematoxylin and eosin
i.p.: Intraperitoneal
IACUC: Institutional Animal Care and Use Committee
INL: Inner nuclear layer
IRL: Inner retinal layer
ICC: interclass correlation coefficient
MBP: Myelin basic protein
MOG: Myelin oligodendrocyte glycoprotein
MS: Multiple sclerosis
OCT: Optical coherence tomography
ON: Optic neuritis
PFA: Paraformaldehyde
PLP: Proteolipid lipoprotein
PT: Pertussis toxin
RGC: Retinal ganglion cell
RNFL: Retinal nerve fiber layer
TCR^2D2^: T cell receptor transgenic mouse specific for MOG_35–55_
TEM: Transmission electron microscopy
TUNEL: Terminal deoxynucleotidyl transferase dUTP nick end labeling
