# Supplementary figures and images for: Monitoring retinal changes with optical coherence tomography predicts neuronal loss in experimental autoimmune encephalomyelitis
Source: J Neuroinflammation. 2019 Nov 4;16:203. doi: 10.1186/s12974-019-1583-4 (PMC6827223; doi:10.1186/s12974-019-1583-4)

## Slide 1
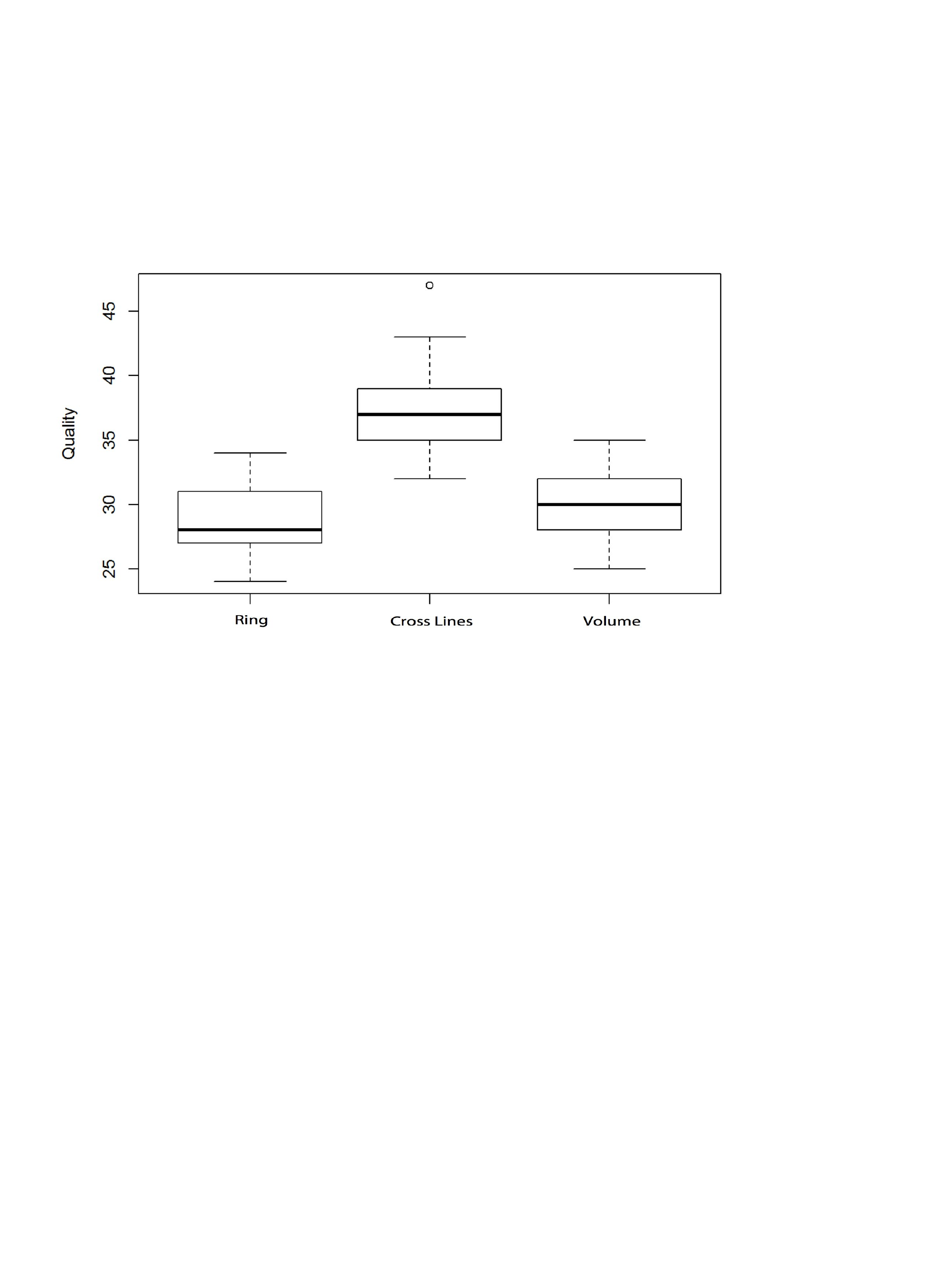

Supplement: Supplementary file 1 — Additional file 1: Figure S1. Quality scores (a measure of signal intensity). Quality above 20 is considered acceptable, quality above 30 is considered excellent. [file 12974_2019_1583_MOESM1_ESM.pptx]

## Slide 1
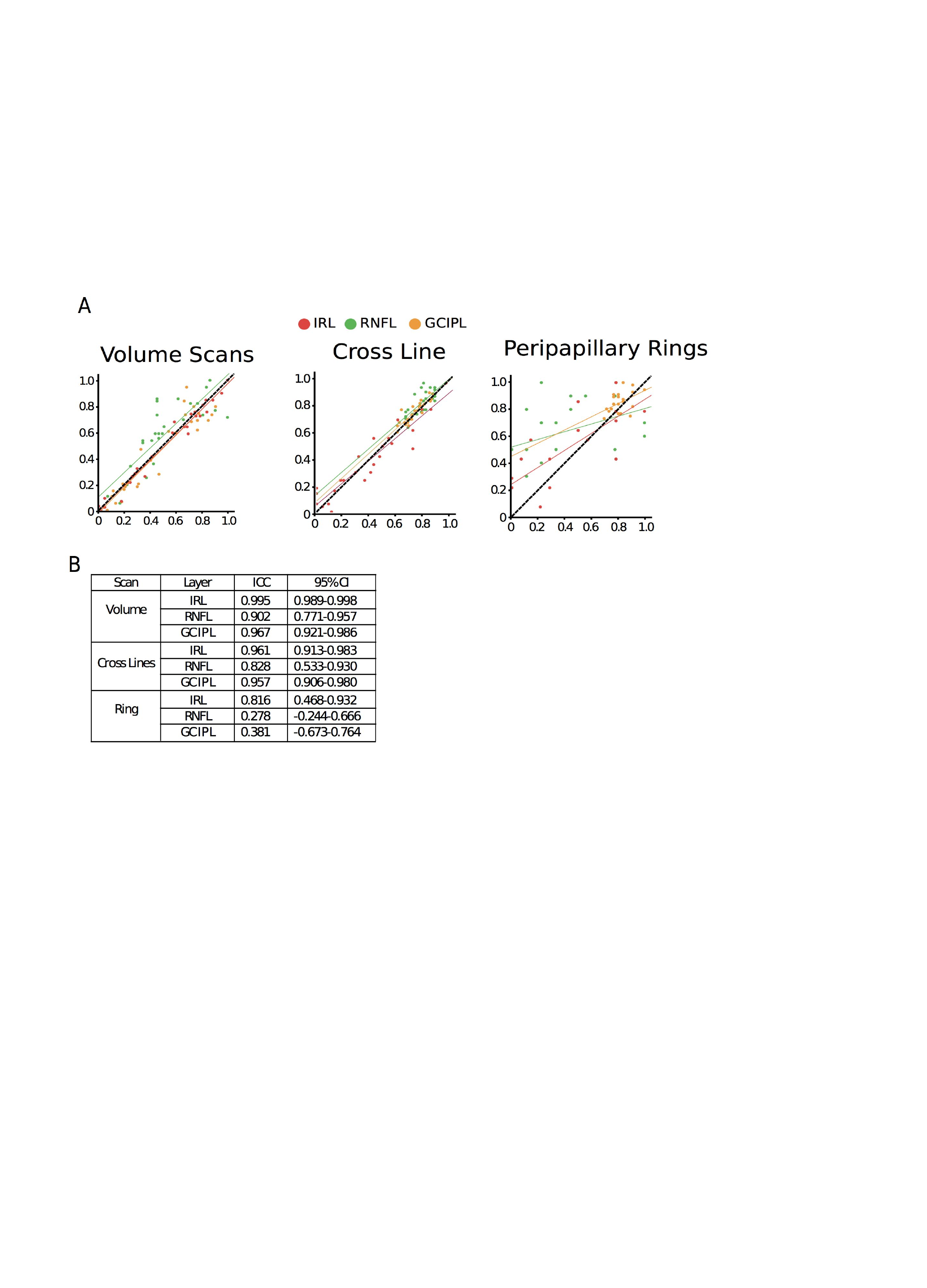

Supplement: Supplementary file 2 — Additional file 2: Figure S2. A: OCT scans segmented by two independent raters with results plotted along a linear regression line. Each point represents a single eye of a mouse. The dotted line the reference for 100% agreement between both raters. Note the relatively improved performance characteristics for volume scans over line scans. B: Interclass correlation coefficients in the different protocols analyzed. [file 12974_2019_1583_MOESM2_ESM.pptx]

## Slide 1
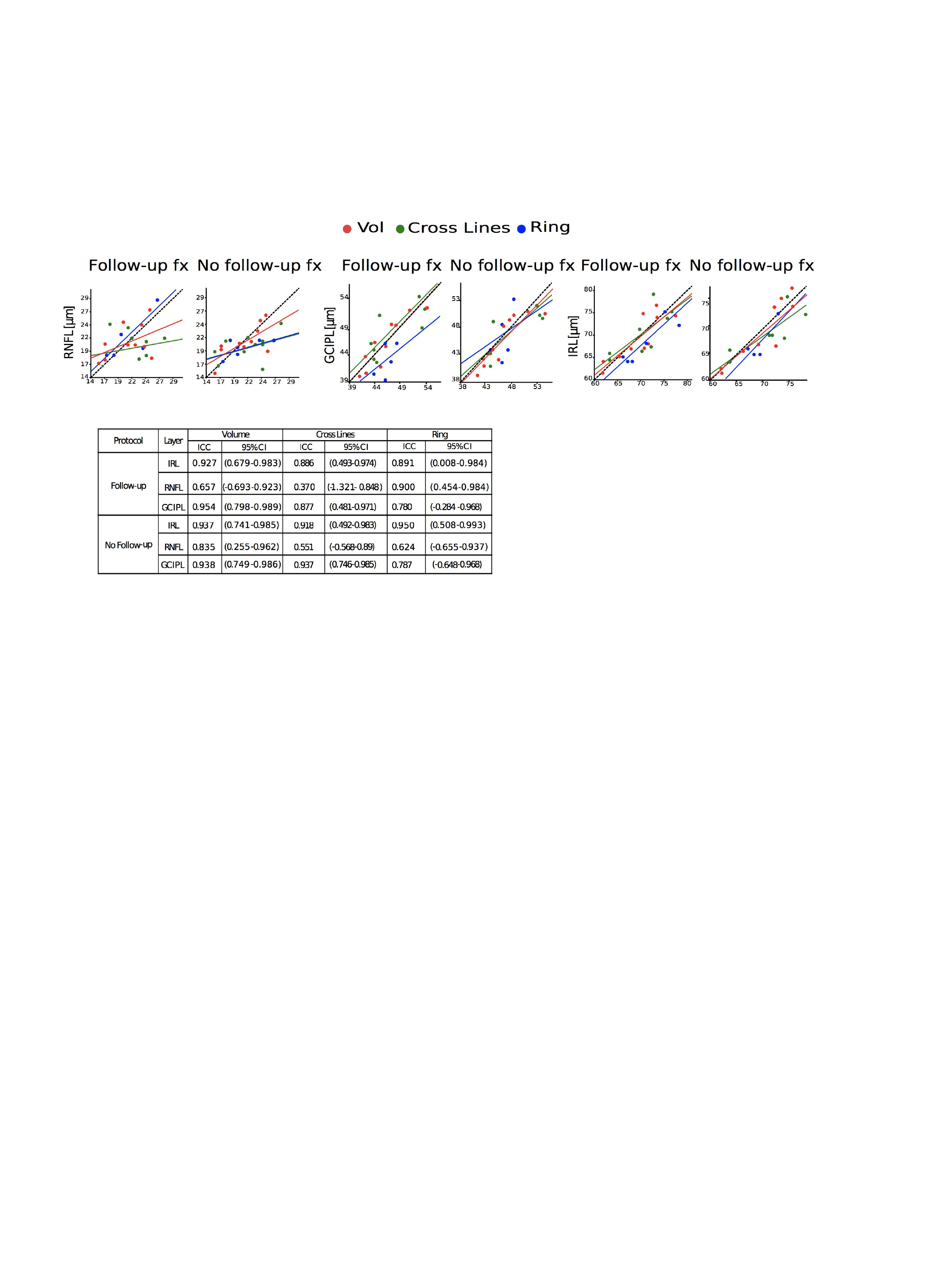

Supplement: Supplementary file 3 — Additional file 3: Figure S3. Serial OCT scans of wild-type mice segmented by a single rater. Note: animals were entirely removed and repositioned between scans. Table demonstrates the interclass correlation for different scan protocols. Note volume scans outperformed line scans and follow-up function adds little benefit to reproducibility. In addition, aggregating layers into either total retinal thickness or inner retinal layers generally outperforms individual layers. [file 12974_2019_1583_MOESM3_ESM.pptx]
